# Outcome of Open Hip Reduction, Pelvic Osteotomy, and Varus Derotational Osteotomy in Children With Cerebral Palsy: A Retrospective Study

**DOI:** 10.7759/cureus.53996

**Published:** 2024-02-11

**Authors:** Ozair Bin Majid, Zayed S Alzayed, Iram Saba, Alia A Aournaser, Ruby Anne A Valoria, Saeed Koaban, Shahad A Zaabi, Alaeldein A Nogud, Abdulrahman M Sharif

**Affiliations:** 1 Orthopaedic Surgery, Sultan Bin Abdulaziz Humanitarian City, Riyadh, SAU; 2 Research, Sultan Bin Abdulaziz Rehabilitation Center, Riyadh, SAU; 3 Radiology, Sultan Bin Abdulaziz Humanitarian City, Riyadh, SAU; 4 Nursing, Sultan Bin Abdulaziz Humanitarian City, Riyadh, SAU; 5 Orthopaedic Surgery, Security Forces Hospital, Riyadh, SAU; 6 Orthopaedics, Sultan Bin Abdulaziz Humanitarian City, Riyadh, SAU

**Keywords:** dega pelvic osteotomy, varus derotation osteotomy vdro, open hip reduction, spastic hip dislocation, cerebral palsy

## Abstract

Introduction

For spastic hip dislocations, a variety of operations are available with open hip reduction and varus derotational osteotomy of the proximal femur combined with pelvic osteotomy ± adductor release being a good option with favourable outcomes. This study aims to assess the outcome and complications of combined open hip reduction with pelvic osteotomy and varus derotational osteotomy.

Methods

In this study, 70 hips in 52 patients with spastic hip dislocation due to cerebral palsy were included. All included patients were treated surgically in our institute between January 2016 and December 2021. There were 31 males and 21 females. For each patient, information was collected about the age at the time of surgery and different radiological parameters at three different time intervals: pre-operatively, immediately post-operatively, and at the final follow-up. We also collected information about any complications arising from the surgery performed.

Results

The mean duration of follow-up was 19.58 months. The acetabular index decreased from an average of 35.01° to 17.18° with a mean difference of 17.83° (p<0.001). The central edge angle, which averaged -49.13° in the pre-operative period, increased to 26.34° and then marginally decreased to 25.47° at the final follow-up. The average migration index of 80.51% in the pre-operative period improved to 1.4% post-operatively with a mean difference of -79.11% (p<0.01). The migration index increased to 8.54% at the final follow-up. Similarly, the neck-shaft angle, which averaged 160.89° in the pre-operative period, decreased to 125.23° at the time of final follow-up with a percentage change of -22.16%.

Conclusion

Single-stage combined surgery in the form of combined open hip reduction and pelvic osteotomy with varus derotational osteotomy successfully treats the condition and shows good outcomes in patients with spastic hip dislocations. This treatment is associated with very few complications.

## Introduction

In patients with cerebral palsy, hip deformity is a common condition encountered, which can range from mild subluxation to complete dislocation of either one hip or both hips. The spastic muscles around the hip joint associated with coxa valga and femoral anteversion result in posterolateral and superior migration of the femoral head [1]. Studies have reported up to 45% incidence of hip subluxation or dislocation in cerebral palsy [2]. Many different methods of management of hip deformity have been suggested. Some of the common procedures performed are soft tissue releases, open hip reduction ± pelvic osteotomy and femoral varus derotation osteotomy (VDRO), and VDRO alone [3,4]. The main goal of surgery in non-ambulatory children is to prevent pain, degenerative changes, and progressive deformity, which can cause severe trouble in maintaining sitting balance and perineal hygiene [5]. In the literature, there is a lot of controversy about whether extensive surgery in the form of open hip reduction ± pelvic osteotomy in addition to VDRO is required in most patients or whether VDRO alone is sufficient in the majority of the patients. There is also a lack of consensus about prophylactic osteotomy of the contralateral uninvolved hip [1,5-7].

The purpose of this study was to evaluate the radiological outcomes of combined open hip reduction with pelvic osteotomy and VDRO in severely involved patients with cerebral palsy. We assume a better outcome with a more comprehensive approach in the form of a combined procedure.

## Materials and methods

This retrospective study was conducted in Sultan Bin Abdulaziz Humanitarian City, Riyadh, Saudi Arabia. The study was approved by the Institutional Review Board of Sultan Bin Abdul Aziz Humanitarian City (approval number: 95-2023-IRB). Patients in the age group of 3-14 years with the diagnosis of cerebral palsy who were non-ambulatory with total involvement, treated with combined open hip reduction, femoral VDRO, and pelvic osteotomy were included. Patients aged less than three years or more than 14 years and patients with other disorders such as spina bifida, arthrogryposis multiplex congenita, myopathies and other syndromes, and genetic disorders were excluded. Patients who were treated previously with another form of procedure, different from our proposed procedure, and/or who were lost to follow-up were also excluded.

**Figure 1:**
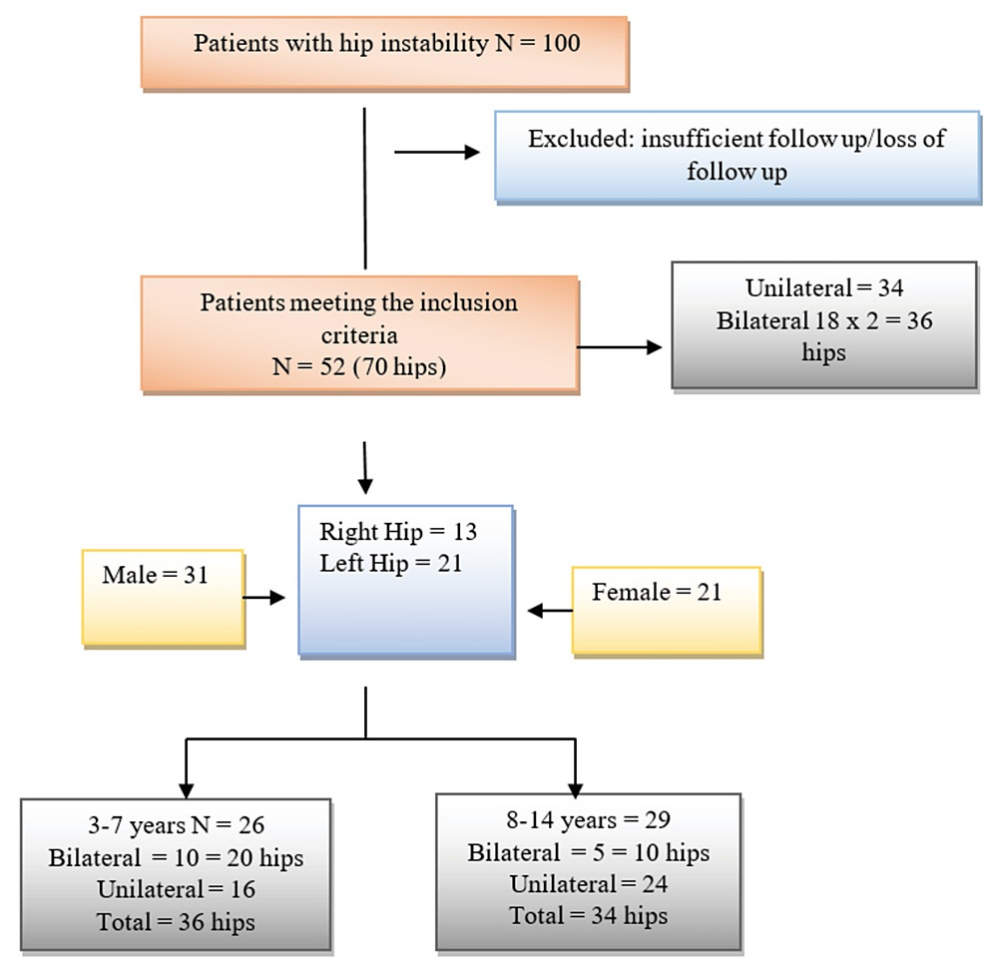
Flow chart of inclusion and exclusion of study patients with cerebral palsy treated with open hip reduction, pelvic osteotomy, and VDRO. VDRO: varus derotation osteotomy

The study initially included 100 patients with hip dislocation treated with combined open hip reduction, femoral VDRO, and pelvic osteotomy from January 2016 to December 2021 at our centre. We finally enlisted 70 hips in 52 patients who met the inclusion criteria. Thirty-four patients had unilateral hip surgeries, with 13 right hip surgeries and 21 left hip surgeries, whereas 18 patients had bilateral hip surgeries. There were 31 males and 21 females (Figure 1). 

For each patient, information such as age in years at the time of surgery and mean follow-up in months was calculated. With the help of our hospital's electronic database, radiological parameters such as acetabular index (AI), central edge angle (CEA), and migration index (MI) were measured pre-operatively, immediately post-operatively, and at the time of final follow-up. Similarly, femoral neck shaft angle (NSA) was also measured pre-operatively and at the time of final follow-up only, and not immediately post-operatively due to the unavailability of immediate post-operative x-ray in true anteroposterior view to measure NSA. Patients were also evaluated for minor and/or major complications.

Pre-operative evaluation

Patient evaluation was done by a multidisciplinary team, including a pediatric orthopaedic surgeon, physiatrist, pediatric gastroenterologist, dietician, and pediatric anesthesiologist. Radiological evaluation was done with a plane radiograph in anteroposterior and frog-leg lateral view.

Surgical technique

Initially, open hip reduction of the dislocated hip was performed through an anterior approach. Then, through a lateral approach to the proximal femur, VDRO was performed, and osteotomy was fixed using an angled blade plate with variable angles ranging from 90° to 110°. Derotation was performed to correct the femoral anti-version. After reducing the hip through a small transverse incision in the groin, adductor tenotomy was done whenever the adductor was found tight. The adductor tenotomy was performed for 89% of hips. Modified Dega-type pelvic osteotomy was then performed using osteotomes, and a tri-cortical iliac crest allograft was placed to correct the acetabular index. After capsuloraphy, the wound was closed over a drain and immobilised in a hip spica cast for six weeks. After the cast removal, a hip abduction splint was applied, and patients were admitted to our centre for a comprehensive rehabilitation program lasting four to six weeks.

Statistical analysis

The data was analysed using IBM SPSS Statistics for Windows, Version 23.0 (Released 2015; IBM Corp., Armonk, New York, United States). The continuous data was presented as mean ± standard deviation and range. The post-surgery and pre-surgery changes in study variables (AI, CEA, and MI) were compared and checked for differences using a paired t-test, and the percentage change was calculated and reported. The figures were plotted in Microsoft Excel 365 (Microsoft Corporation, Redmond, Washington, United States).

## Results

The mean age at the time of surgery was 7.85 years (range, 3-14). Patients with bilateral hip surgery were operated on at different time intervals. A total of 36 hips were operated on in the age range of three to seven years and 34 hips were operated on in the age group of 8-14 years. The mean follow-up was 19.58 months (range, 6-72). The radiological parameters at the baseline were: AI ranged from 22° to 65° with mean AI at 35.01°, CEA ranged from -154° to 14° with a mean of -49.13°, MI ranged from 8% to 100% with a mean of 80.51%, and finally, NSA had a range of 131° to 180° with a mean of 160.88° (Table 1). 

**Table 1:** Baseline characteristics of the patients.

Variable	Min	Max	Mean ± SD
Mean age (years)	3	14	7.85 ± 3.36
Mean follow-up (months)	6	72	19.58 ± 14.12
Mean acetabular index (^0^)	22	65	35.01 ± 8.89
Mean central edge angle (^0^)	-154	14	-49.13 ± 47.57
Mean migration index (^0^)	8	100	80.51 ± 24.94
Mean neck shaft angle (^0^)	131	180	160.88 ± 10.53

The immediate post-operative x-rays showed that the AI decreased from an average of 35.01° to 17.18° with a mean difference of -17.83° (p<0.001). However, with time the AI increased marginally, with the final AI reaching 20.73° with a mean difference of +3.89° (p-value 0.0013). Similarly, the CEA, which averaged -49.13° in the pre-operative period, increased to 26.34° and then marginally decreased to 25.47° at the final follow-up. The average MI of 80.51% in the pre-operative period improved to 1.4% post-operatively with a mean difference of -79.11% (p<0.01). This MI increased to 8.54% at the final follow-up. No significant difference was noted in the trends in the age groups of 3-7 years and 8-14 years (Tables 2, 3).

**Table 2:** Pre- and post-surgery changes in acetabular index, central edge angle, and migration index among different age groups. p-value <0.05 was considered statistically significant.

Variable		Pre-Surgery	Immediate Post-Surgery	Mean Difference	P-value
Acetabular index(º)	3-7 years	34.39 ± 9.28	15.56 ± 7.22	-18.83	< 0.01
	8-14 years	35.68 ± 8.71	18.91 ± 5.40	-16.76	< 0.01
	Overall	35.01 ± 8.90	17.19 ± 6.52	-17.83	< 0.01
Central edge angle(º)	3-7 years	-34.35 ± 38.20	25.16 ± 10.48	59.52	< 0.01
	8-14 years	-64.79 ± 51.46	27.58 ± 8.12	92.38	< 0.01
	Overall	-49.13 ± 47.57	26.34 ± 9.42	75.48	< 0.01
Migration index MI(%)	3-7 years	77.86 ± 22.57	1.13 ± 4.32	-76.72	< 0.01
	8-14 years	83.32 ± 27.29	1.67 ± 4.25	-81.65	< 0.01
	Overall	80.51 ± 24.94	1.4 ± 4.26	-79.11	< 0.01

**Table 3:** Immediate post-surgery and final follow-up changes in acetabular index, central edge angle, and migration index among different age groups. p-value<0.05 was considered statistically significant.

Variable		Post-Operative	Follow-up	Mean Difference	P-value
Acetabular index(º)	3-7 years	15.56 ± 7.22	19.44 ± 6.26	3.89	0.0026
	8-14 years	18.91 ± 5.4	22.09 ± 6.25	3.18	0.0004
	Overall	17.19 ± 6.51	20.73 ± 6.31	3.54	0.0013
Central edge angle (º)	3-7 years	25.16 ± 10.64	24.44 ± 10.51	-0.72	0.8746
	8-14 years	27.58 ± 8.12	26.55 ± 14.35	-1.03	0.6021
	Overall	26.34 ± 9.42	25.47 ± 12.46	-0.87	0.6420
Migration index MI(%)	3-7 years	1.13 ± 4.32	10.11 ± 14.58	8.98	< 0.0001
	8-14 years	1.67 ± 4.25	6.88 ± 9.60	5.21	< 0.0001
	Overall	1.4 ± 4.26	8.54 ± 12.43	7.14	< 0.0001

**Figure 2:**
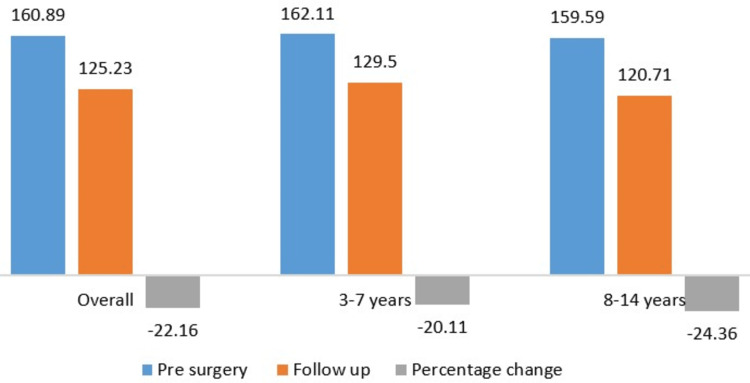
The change in neck-shaft angle pre-surgery and at the final follow-up and percentage difference among different age groups.

**Figure 3:**
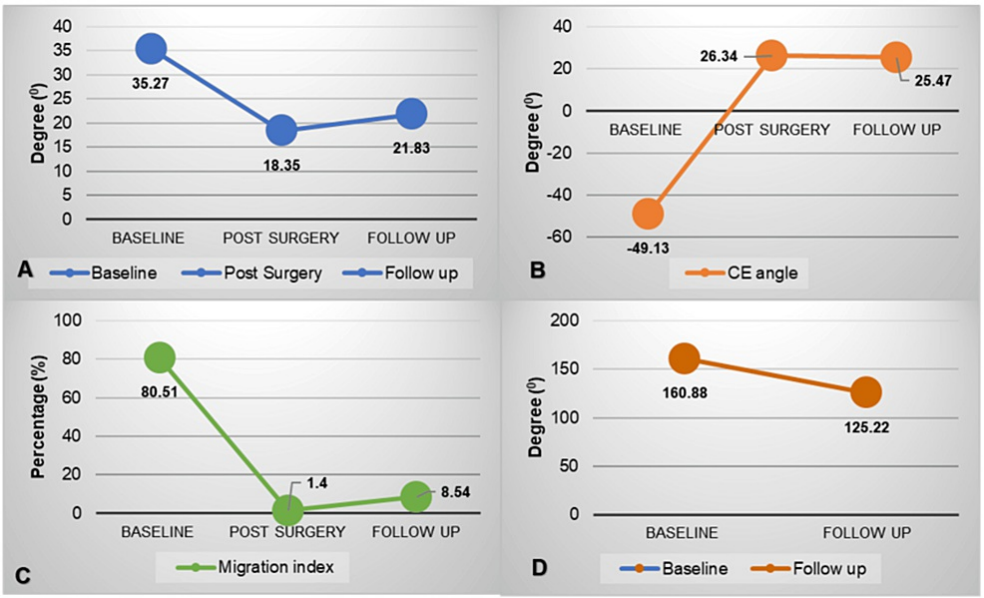
The overall change in radiological parameters at the baseline, immediate post-surgery, and at final follow-up (A) Change in the acetabular index in degrees; (B) Change in the central edge angle in degrees; (C) Change in the migration index in percentage; (D) Change in the neck shaft angle in degrees

The NSA, which was measured only in the pre-operative period and at the final follow-up, showed significant improvement. NSA averaged 160.89° in the pre-operative period and decreased to 125.23° at the final follow-up with a percentage change of -22.16% (Figure 2). The overall change in different radiological parameters over time is depicted in Figure 3.

**Figure 4:**
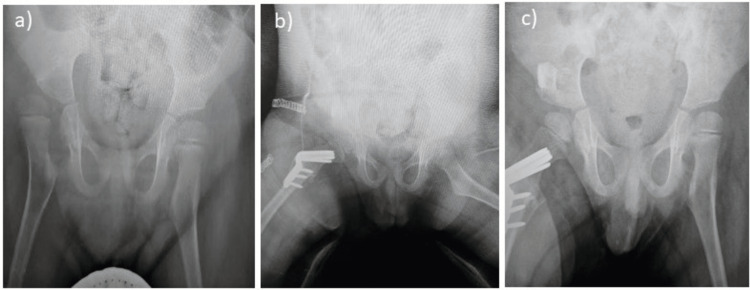
Plain x-rays of a six-year-old patient with right hip dislocation (a) pre-operative x-ray; (b) x-ray of the same patient immediately after surgery with reduced right hip; (c) x-ray of the same patient at the 20-months follow-up showing a reduced hip joint.

**Figure 5:**
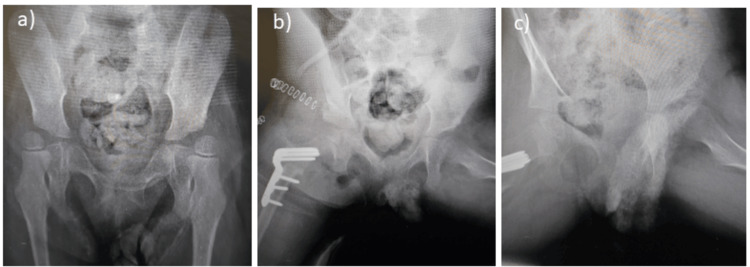
Plain x-ray of a five-year-old patient with right hip dislocation (a) Pre-operative x-ray; (b) x-ray of the same patient immediately after surgery with reduced right hip; (c) x-ray of the same patient at the 22-month follow-up showing a reduced hip joint.

Figure 4 and Figure 5 show the pre-operative, immediate post-operative, and final follow-up x-rays of a six-year-old and five-year-old patient, respectively.

**Figure 6:**
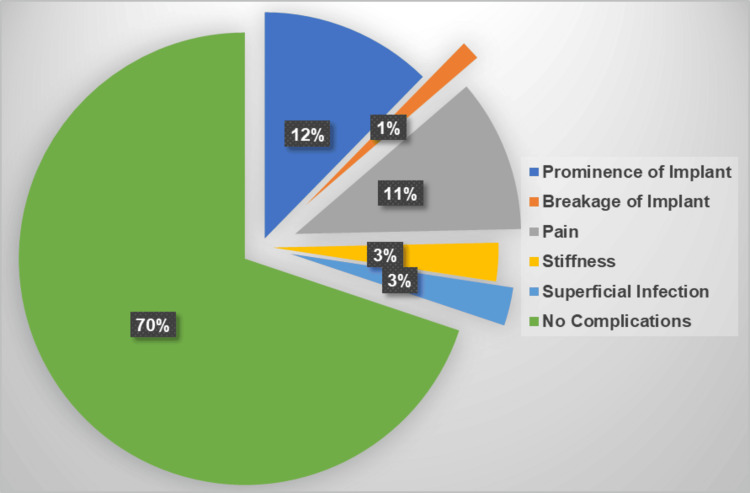
The percentage of complications; 70% of patients did not have any post-operative complications.

The distribution of complications is depicted in Figure 6, which shows that 70% of patients presented without any post-operative problems, 12% of patients displayed prominence of the implant, and 11% of patients experienced pain.

## Discussion

Although various treatment options, such as soft tissue releases and VDRO alone or in combination with open hip reduction and simultaneous pelvic osteotomy, have been advised with the aim of improving hip stability for patients with spastic hip dislocations [8-12], only a few studies have reported radiographic outcomes in patients with spastic hip dislocations who underwent more extensive procedures in the form of open hip reduction with VDRO in combination with pelvic osteotomy and soft tissue release.

We studied the outcome of a single-stage combined procedure of open hip reduction, VDRO, and pelvic osteotomy ± adductor release in patients with spastic hip dislocations. Various pelvic osteotomies can be performed to correct acetabular dysplasia [11,13]. We performed a modified type of Dega osteotomy to achieve a concentric reduction and cover the acetabular deficiency.

Al-Ghadir et al., in a retrospective study, analysed 52 hips in patients with spastic cerebral palsy treated from January 1997 to January 2007 [5]. Patients were divided into two groups: Group A had combined surgery and Group B was treated with VDRO alone. Radiological parameters such as CEA, MI, AI and NSA were studied. According to the authors, with a combined approach, there was a significant decrease in pain and improvement in CEA and AI in patients. They concluded that the clinical and radiological results were much better in a combined approach than in VDRO alone, thus justifying a more extensive approach. In line with this, the current study showed significant improvement in all radiological parameters. Furthermore, none of our patients required revision procedures.

Mubarak et al. studied 18 spastic hip subluxations or dislocations treated with combined surgery [9]. They noticed that 17 of 18 hips remained anatomically reduced at a mean follow-up of six years and 10 months. Huh et al. performed a retrospective study of 116 hips treated surgically with VDRO alone and VDRO combined with open hip reduction and/or pelvic osteotomy [1]. Postoperative radiographic parameters were similar in the two groups, and there was no difference in complications between the two groups. They concluded that combined procedures should be reserved for high MI. However, they added that as the patients were very young at the time of final follow-up, these results only showed the medium-term effectiveness of VDRO alone without additional pelvic surgery.

Many studies which are in favour of limited surgery have either a short-term follow-up or the patients are at a relatively young age at the time of final follow-up, thus increasing the possibility of re-dislocation in future. In our short-term follow-up, we did not encounter any case of re-dislocation that required revision surgery. Shore et al. suggested that in patients with high Gross Motor Function Classification System (GMFCS) scores, VDRO alone is insufficient and can result in revision hip surgery [14]. Poul et al., in their retrospective study of 61 hips in 50 patients, found that VDRO alone was less effective in providing hip joint stability and more tilting of the proximal fragment of the femur was necessary to achieve a good reduction in VDRO alone [15]. They further concluded that combination surgery provided better outcomes in treating unstable hips. In the current study, we achieved concentric reduction and good coverage of the femoral head with an extensive approach.

Park et al. in their study of 144 patients who underwent VDROs, with or without open hip reduction, studied the outcomes based on various radiological parameters [16]. They noticed satisfactory outcomes in 78.5% of hips. Furthermore, they concluded that the preoperative AI did not affect the outcome. According to the authors, the thresholds of pre- and post-operative MI can determine the need for a concomitant pelvic osteotomy.

In another retrospective study, conducted by Oto et al., combined VDRO and Dega osteotomy were performed in 22 hips, and the radiologic parameters such as MI, AI, and NSA were measured [17]. The mean follow-up period was 36.1 months. Oto et al. noted that the mean AI was 33.2° preoperatively and 20.4° postoperatively. In the current study, the mean pre-operative AI was 35.01°, which reduced to 17.18° postoperatively and then had a marginal increase to 20.73° at the final follow-up. Similarly, in the study by Oto et al., the MI and NSA were 72.7% and 160°, respectively, which improved to 24.3% and 130°, respectively, post-operatively. In our study, the MI improved from 80.51% pre-operatively to 1.4% post-operatively and then marginally deteriorated to 8.54% at the final follow-up. Furthermore, in our study, the pre-operative NSA improved from 160.89° to 125.23° at the final follow-up. Our mean follow-up was 19.58 months.

In the literature, the complication rates after hip surgeries in patients with cerebral palsy vary considerably, ranging from 0% to 81% [18-22]. Al-Ghadir et al. found that the risk of complications is related to the severity of the disease [5]. Furthermore, in the same study, none of the patients developed delayed unions, avascular necrosis of the femoral head, or post-operative infection. Similarly, Gordon et al., in a study of 52 hips that underwent combined surgery, found no cases of re-dislocations, subluxation, or avascular necrosis at three three-year follow-ups [7]. In our study, complications were seen in 30% of the patients, including pain, implant prominence, implant breakage, stiffness of the joint, and superficial infection. None of our patients developed avascular necrosis of the head, re-dislocation of the hip or deep infection requiring removal of implant, and debridement.

We studied the outcome of surgery in two age groups (three to seven years and 8-14 years) and documented all the pre-operative and post-operative radiological parameters in both age groups. In the review of similar studies, none were found to have studied the effect of surgery and its outcome in two different age groups. The limitation of our study was a short follow-up duration. Our study also had a small sample size, and retrospective studies are open to many types of bias affecting the results. Despite the limitations, our results were promising and very useful in drawing a lot of meaningful interferences. We recommend that further studies take these limitations into consideration. 

## Conclusions

Different surgical treatment options are being utilized to treat spastic hip dislocation in cerebral palsy. In the current study, the surgical process of open hip reduction and VDRO combined with pelvic osteotomy ± adductor release has been seen to improve the clinical and radiological outcome in the majority of the patients. It helps in achieving a well-positioned, supple, and painless hip joint. The findings of the present study may be of particular interest to pediatric orthopaedic surgeons due to the good outcome of the procedure and very low complication rates. 
